# Using the Job Burden-Capital Model of Occupational Stress to Predict Depression and Well-Being among Electronic Manufacturing Service Employees in China

**DOI:** 10.3390/ijerph13080819

**Published:** 2016-08-12

**Authors:** Chao Wang, Shuang Li, Tao Li, Shanfa Yu, Junming Dai, Xiaoman Liu, Xiaojun Zhu, Yuqing Ji, Jin Wang

**Affiliations:** 1 National Institute of Occupational Health and Poison Control, Chinese Center for Disease Control and Prevention, Beijing 100050, China; wchao1987@126.com (C.W.); lxm1926@hotmail.com (X.L.); happyzhuxj@163.com (X.Z.); jyq915204131@163.com (Y.J.); jinw1003@hotmail.com (J.W.); 2 Graduate School of Chinese Center for Disease Control and Prevention, Beijing 102206, China; 3 Henan Provincial Institute for Occupational Health, Zhengzhou 450052, China; yu-shanfa@163.com; 4 School of Public Health, Fudan University, Shanghai 200032, China; jmdai@fudan.edu.cn

**Keywords:** occupational stress, job burden—capital model, structural equation model, depression, well-being

## Abstract

*Background*: This study aimed to identify the association between occupational stress and depression-well-being by proposing a comprehensive and flexible job burden-capital model with its corresponding hypotheses. *Methods*: For this research, 1618 valid samples were gathered from the electronic manufacturing service industry in Hunan Province, China; self-rated questionnaires were administered to participants for data collection after obtaining their written consent. The proposed model was fitted and tested through structural equation model analysis. *Results*: Single-factor correlation analysis results indicated that coefficients between all items and dimensions had statistical significance. The final model demonstrated satisfactory global goodness of fit (CMIN/DF = 5.37, AGFI = 0.915, NNFI = 0.945, IFI = 0.952, RMSEA = 0.052). Both the measurement and structural models showed acceptable path loadings. Job burden and capital were directly associated with depression and well-being or indirectly related to them through personality. Multi-group structural equation model analyses indicated general applicability of the proposed model to basic features of such a population. Gender, marriage and education led to differences in the relation between occupational stress and health outcomes. *Conclusions*: The job burden-capital model of occupational stress-depression and well-being was found to be more systematic and comprehensive than previous models.

## 1. Introduction

With further industrialization and modernization, increasing occupational psychological problems have gained attention from all social sectors. Indeed, occupational stress can lead to negative conditions such as exhaustion [1] and depression [2] and might seriously damage the occupational population’s work ability, social function and status of well-being [3,4]. Meanwhile, depression has become one of the most common psychological disorders worldwide. The World Health Organization (WHO) ranked it as the fourth cause of disability, but predicted it to increase to second place by 2020. In China, depression has been ranked as the second highest burden of medical expenses [5]. Now, the occupational population is the basis of social and economic development, and maintaining their safety and health is an important factor for social progress [6]. Depression affects people’s ability to work, possibly leading to low production efficiency and even disability. In contrast, well-being is another important guarantee for their safe and efficient work. Thus, discovering the role of occupational stress in maintaining well-being and development of depression has been the focus in occupational health studies. Some studies have shown that occupational stress influences depression and well-being through various working conditions, i.e., effort-reward imbalance is closely associated with depression; job demands (e.g., high work pressure, emotional needs, role ambiguity) might lead to sleep disorders [7], anhedonia [8], and so on, which serve as the main component of depression. Well-being and protective factors, specifically, social support [9], self-esteem [10] and autonomy [11] might alleviate occupational factors’ negative effects through increasing employee learning opportunities [12,13] and improving a sense of integration in the work [14]. 

At present, most research on this topic has been conducted on health hazards and thereby achieved great success [11,12,13]. However, to predict and evaluate occupational stress and its outcomes, most studies are based on occupational stressors, or, in recent years [12,15,16,17], they have used two internationally recognized models, i.e., the job demands-control (JDC) model [15] and the effort-reward imbalance (ERI) model [16]. These two models’ simple structure and content have become the greatest advantage for their application, and they have been widely used in the study of occupational stress for the past 30 years. However, this simplicity also results in prediction or evaluation bias for occupational stress and its outcomes. The models focus on two important parts of occupational stress that have been respectively taken as the theoretical basis of its prediction and evaluation. However, in the face of the occupational population’s growing complexity and occupational characteristics, these evaluation results might, to some extent, not reflect the current occupational population’s panorama of occupational stress [12], leading to uncertain correlations with health outcomes. In other words, stress evaluation by JDC or ERI models alone might miss certain populations’ stressed status. For example, the ERI questionnaire cannot accurately assess psychological demands on visual display terminal operators, thus potentially drawing biassed conclusions on occupational stresses’ prevalence. Many works based on the two models have shown many inconsistent results, and job complexity leads, possibly, to producing one-sided evaluation results through application of the two single models [18,19]. Also, many studies have questioned how systematic the two models actually are [20].

For deeper understanding of the JDC and ERI, many studies have conducted research on occupational stress by using the JDC and ERI simultaneously to solve occupational stress problems under more comprehensive and systematic conditions. Obviously, however, one issue cannot be avoided even by using these two models simultaneously: their evaluation systems are independent. In addition, they were initially designed to evaluate occupational stress from independent angles, and confounding their dimensions or items without a theoretical basis is inadvisable. Although simultaneous JDC and ERI usage can measure stress factors from different perspectives, it is difficult to explain them according to one comprehensive index in a common framework, especially for laypeople. Thus, simultaneous usage maintains just the role of simultaneousness. To compensate for this drawback and for extensive research on occupational stress, we require development of a theoretical platform. 

In fact, some scholars have already noticed the issue and made efforts to resolve it. In 2003, Bakker et al. [20] proposed the “Job demand–resource model”. This model attempted reforms in the working condition of job demands and introduced “job resource” as the dimension interactive with “job demand”. Subsequent literature has shown this model’s practicability and applicability [12]. As a model recently gaining more and more attention, the job demand-resource model also indicates that measurement of psychological working conditions needs to be comprehensive. Otherwise, it might lead to biassed assessment. However, after reviewing the literature regarding the demand-resource model, we have found that only some of the model’s scales/items are selected from occupational stress-related measurement tools, while items’ specificity to stress measurement remains to be discussed. At the same time, we found that various studies have selected different scales for establishing the demand-resource model’s framework, scales not even familiar in the study of occupational stress. In an article on the relationship between the job demand-resource model and burnout, Bakker himself also indicated that the model’s scales have been selected from heterogeneous scales without the assessment of their reliability and validity. Therefore, we believe that the demand-resource model might have disadvantages in the specificity of measuring occupational stress. 

By considering these issues, and for developing a model that fits the Chinese social and cultural background, we propose the “job burden-capital model” on the basis of the widely used JDC and ERI in China for predicting and evaluating the occupational population’s health outcomes (depression and well-being). The model’s basic theory supposes that when the job burden-capital does not match, since the occupational population’s job burden goes beyond the capital owned, risk of depression increases. Additionally, regulation of personal characteristics affects the extent and speed of depression’s development. 

“Job burden” mainly refers to those psychological, physical, organizational, social and personal job factors that require enhanced or transitory physical and/or psychological diathesis or capability and lead to daily costs physiologically and/or psychologically. For example, the work environment, task intensity, psychological burden, skill requirements and so on probably increase risk of depression. When the occupational staff’s available work capital is certain, high job burden might directly increase risk for and development of depression [21]. It might also reduce their well-being and work efficiency [22]. 

“Capital” refers to those psychological, physical, organizational, social and personal factors that can function to address the requirement of job burden, fulfil job tasks, alleviate depressed feelings [12], increase sense of accomplishment and improve individuals’ ability. Examples are high personal job skills, job autonomy, fair treatment, future development, work stability, work identity, sense of respect, income and social support. Enough capital is an important guarantee of occupational groups fulfilling occupational tasks; capital is directly related to their well-being [23]. 

In addition, employees’ depression and well-being might also be regulated by differences in individual personality [24], especially by the trait of inability to withdraw from obligations at work [25,26,27]. For example, people who are too concerned about work might be more sensitive to the reaction of depression than others. Job burden and capital should be matched in professional life to ensure the staff’s completion of assignments and their well-being and satisfaction. 

The present study explores a more comprehensive and systematic association between occupational stress and depression or well-being by proposing the innovative model of job burden-capital. Three hypotheses are to be confirmed: (1) Job burden and capital directly relate to employees’ depression and well-being; (2) Personality can mediate the relationship between job burden-capital and related health outcomes; (3) For occupational groups, the theoretical model has general applicability among populations with different characteristics, and this buffers effects on the model’s association of variables.

## 2. Experimental Section

### 2.1. Population and Investigation Process

This cross-sectional study recruited staff of the electronic manufacturing service industry in Hunan Province, China, as participants. Before the field survey, the Chinese Center for Disease Control and Prevention organized the study group, united with the Provincial Prevention and Treatment Center of Occupational Disease, communicated with companies to be surveyed, and informed them of the investigation’s purpose and significance. Upon consent of the local unit, we conducted on-site mobilization. Formal investigation was officially conducted from June 2015 to July 2015. Investigators with unified training conducted the field survey, and participants completed the questionnaire on the spot, with recovery after the audit. Inclusion criteria for investigation participants were as follows: (1) Participants had worked continuously for 6 months or more in the position; (2) there was no history of mental illness and no history of psychotropic drug use for one week before the investigation; (3) there was no long-term sick-leave history; (4) participants completed on-site mobilization and voluntarily participated in the survey upon informed consent. The Medical Ethics Committee of the Institute of Occupational Health and Poison Control of the Chinese Center for Disease Control and Prevention approved this study.

A total of 1800 questionnaires were issued, 1618 questionnaires were recovered—an effective recovery rate of 89.9%. Participants were 835 males (51.6%) and 783 females (48.4%); their average age was 28.84 ± 6.37 years, and the distribution was uniform. Proportions of the 25-year-old age group, the 26–30 age group and the group aged above 30 were 34.7%, 31.7% and 33.6%, respectively. As for marital status, 981 people (60.6%) were married. The level of education was relatively low as a whole; 441 (27.3%) people had a junior high school education degree, and 809 (50.0%) had a high school/secondary school education degree. Working on an assembly line were 768 people (47.5%), 479 were not on an assembly line (29.6%), and 371 worked in logistical and administrative posts (22.9%).

### 2.2. Hypotheses and Construction of Job Burden-Capital Matching Model

#### 2.2.1. Measurement Method of Each Model Dimension

**Figure 1:**
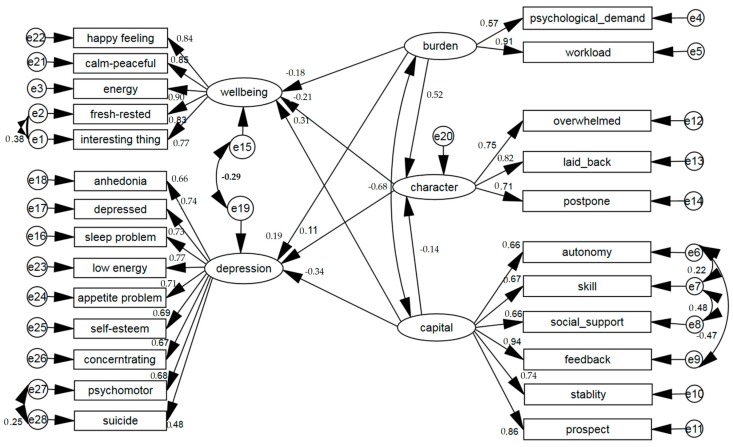
Structural equation model of Job Burden-Capital-Depression-Wellbeing.

The core hypothesis of job burden-capital is that the occupational population’s occupational characteristics, the operational environment and organization and management factors jointly influence occurrence and development of occupational stress, which can be attributed to two working conditions, job burden and capital. Job burden was specifically divided into workload and psychological demands. Capital covers six dimensions: job autonomy (e.g., decision autonomy, independent arrangement), job skills (e.g., skills learning, skills improvement), social support (e.g., peer support, supervisor support), work feedback (e.g., sense of respect, fair treatment, identity, income), work stability and development prospects. In addition, personal characteristics might regulate the degree of occupational stress, so the dimension of individual personality was regarded as a mediating dimension. The model diagram can be seen in Figure 1.

Measurement of all factor items was based on Chinese versions of the job content questionnaire [28] and the effort-reward imbalance questionnaire [29]; the most representative items consistent with the model’s hypothesis and China’s social background were selected. Assessment scales of two Chinese versions of occupational stress developed and optimized by Li Jian in 2004 have wide application in the study of occupational stress in China [30]. Except for some items, such as sense of monotony, which are not in line with the current Chinese economic situation and the model’s hypothesis, or which are redundant, the main dimensions remain to be assessed. Moreover, qualitative interviews and pre-surveys have been conducted before the development of measuring items, and items that were not adapted to the cultural context have been modified. Thus, the language context is more in line with speaking habits and the traditional thinking mode of the Chinese. All items adopt five levels of Likert ratings (1 = completely disagree to 5 = completely agree); this unified rating strategy made ratings more consistent and thus ensures data’s consistency and meaningfulness.

#### 2.2.2. Job Burden

*Workload*: Workload is based on effort dimensions of effort-reward imbalance model that covers six items, and the measurement and evaluation involves time urgency, work disruption, work responsibility, work overtime, physical demands and increasing job requirements, such as “due to the heavy work load, I always feel that I don’t have enough time”. 

*Psychological demand*: The psychological demand is based on the emotional job demand dimension of the job content questionnaire [31] that covers four items: sense of responsibility, sense of making efforts, sense of urgency and so on, for instance, “I feel it was very fast-paced, and I am unable to stop for a rest”.

#### 2.2.3. Capital

*Autonomy:* Two items were measured, namely, degree of autonomy as in “what to do” and “how to do it”. They are “I have the freedom to decide how to work” and “I have the freedom to decide what work to do”, respectively.

*Skills:* Three aspects were measured, the level of work skills, skills improvement and learning or creativity, for instance, “I can integrate my creativity into my work”.

*Social support:* The most representative questions on family support, support of colleagues or leadership in the social support dimension in the JCQ scale were selected, for example, “I obtain support from my colleagues at work” and “I get along well with my supervisor”.

*Feedback:* The items of the “reward” dimension in the ERI scale were selected, including respect, fairness, recognition, income and so on; six items were measured, for instance, “my income and my effort or performance does not match”.

*Work stability:* Two items for measurement were selected: “My work stability is poor” and “There are unnecessary changes to my work”.

*Work prospect:* Two items for measurement were selected: “My promotion prospects are not promising” and “My work prospects are not consistent with my efforts”.

#### 2.2.4. Personality

The measurement of personality is based on “over-commitment” in the effort-reward imbalance scale, which is described as a “personality trait mainly characterized by the inability to withdraw from work obligations” [32]. The dimension covers three items on work-related content of personality: easily overwhelmed by time pressure, work still in mind before going to sleep (trouble being “laid-back”), and postponing working demands. A specific question includes “I begin to think about work as soon as I get up in the morning”.

#### 2.2.5. Depression

The Patient Health Questionnaire (PHQ) is a self-administered version of the diagnostic instrument for common mental disorders. The PHQ-9 [33] which is the 9-item depression module from the full PHQ is used for evaluation of depression among the study population. The PHQ-9 score ranges from 0 to 27, since each of the nine items can be scored from 0 (not at all) to 3 (nearly every day). The scale has been confirmed to have high reliability and validity [33]. 

#### 2.2.6. Well-Being

The study population’s well-being is assessed by WHO-five well-being scale (WHO-5). Five statements presented (I have felt cheerful and in good spirits; I have felt calm and relaxed; I have felt active and vigorous; I have felt fresh and rested; My daily life has been filled with things that interest me) were assessed on a six-point scale (from never to always), with the possible total score varying from 0 to 25. Higher scores indicate better well-being [34]. 

#### 2.2.7. Statistical Processing

First, we introduced Cronbach’s α to test the internal consistency reliability of the selected items, in order to explore the suitability of including the research variables in the model. Second, Pearson’s test of correlation analysis was conducted to explore relationships between working conditions and health outcomes; for inspection of the model of job burden-capital and health outcome, the structural equation model was adopted to conduct confirmatory factor analysis to test the theoretical framework within the data collected. For goodness-of-fit, the adjustment fitting goodness indicator (AGFI), non-normalised fit index (NNFI), incremental fit indicator (IFI) and root mean square error of approximation (RMSEA) were employed. References show that the model fit coefficient is >0.9, and RMSEA < 0.08 [35], which can be accepted as good fit. According to the Figure 1 model diagram, this study divided working conditions into two latent variables as a whole, namely, job burden and capital, and evaluated the two measured variables of work load and psychological demands after centralization; individual personality was included in the model as an internal adjustment variable. To explore association effects of personality characteristics in the relationship between working conditions and health outcomes (depression and well-being), we conducted an analysis of the mediating effect of the structural equation model [36]. The bootstrap statistical method was used, and the sampling number was set as 5000, according to Hayes [37], taking the bias correction interval as the confidence interval of mediating effect [37]. Epidata 3.1 (“The EpiData Association”, Odense, Denmark) was used for data entry, and SPSS Statistics 19.0 and SPSS AMOS 21.0 (SPSS Inc., Chicago, IL, USA) were used for statistical analysis; α takes 0.05 with two tails. 

### 2.3. Ethics Review and Approval

The study protocol was approved by the Medical Ethics Committee of the National Institute of Occupational Health and Poison Control (Code No. 201502). All participants signed written informed consents.

## 3. Results

### 3.1. Analysis of Correlation and Internal Consistency

Single-factor correlation analysis shows that the correlation coefficients of the three dimensions of job burden, capital and personality are of statistical significance, −0.495, 0.513 or −0.415, respectively. Coefficients of job burden, capital or personality and depression are 0.366, −0.506 or 0.325 respectively with statistical significance, and the coefficients of the three aspects above and well-being are also statistically significant (−0.451, 0.516 and −0.419, respectively). The correlation between depression and well-being is −0.450 (*p* < 0.05). Each of the three dimensions significantly correlated with their sub-items. For example, the relevant coefficients of job burden and workload, and psychological demands are 0.874 and 0.867, respectively. Correlations of sub-items of the three dimensions of job burden, capital and personality also have statistical significance (see Table 1 for details). In addition, results of the internal consistency test shows that Cronbach’s α is between 0.750 and 0.943. Among these, the capital dimension has the highest reliability (0.943), the sub-item of job prospects (0.750) and the sub-entry of work stability have the lowest (0.754), and the internal consistency of remaining dimensions or sub-items are all above 0.8, thus reaching a high level (see Table 1 for details). 

### 3.2. Goodness of Model Fit

After verifying that the selected items and dimensions have correlation and internal item consistency meets the requirements of model fit, we used the structural equation model to conduct data fitting and optimization for model hypotheses; and finally, we verified whether the hypotheses have been established. First, we conducted analysis of the structural equation model to the two basic dimensions of job burden-capital model. Results show that the goodness-of-fit of the modified model is acceptable, compared with the original model (CMIN/DF = 5.18, AGFI = 0.971, NNFI = 0.984, IFI = 0.992, RMSEA = 0.051), which all reach standards of goodness-of-fit indices. After adding the personality dimension into M2, the model also achieves high global goodness-of-fit. On the basis of M2, we added the latent variable of depression as the common outcome variable of three dimensions, and the interactive path among three dimensions was not added. The shows the model’s goodness-of-fit is poor even after modification (CMIN/DF = 14.16, AGFI = 0.836, NNFI = 0.849, IFI = 0.871, RMSEA = 0.090). Similarly, when three dimensions are added to the one-way path of well-being, the goodness-of-fit is unacceptable. Then, we added corresponding paths between three dimensions on the basis of M3, and the result shows that the goodness-of-fit reaches a higher level (CMIN/DF = 6.37, AGFI = 0.919, NNFI = 0.938, IFI = 0.948, RMSEA = 0.058). M6 showed similar results. In M7, we combined M5 and M6, i.e., depression and well-being were included into the model simultaneously with the corresponding paths between the three dimensions at the same time. Then the modified model’s goodness-of-fit reaches a high degree (CMIN/DF = 5.72, AGFI = 0.910, NNFI = 0.940, IFI = 0.948, RMSEA = 0.054). In the final M8 model, we further added the correlation path of depression and well-being, so the final model has the most satisfactory goodness-of-fit (CMIN/DF = 5.37, AGFI = 0.915, NNFI = 0.945, IFI = 0.952, RMSEA = 0.052), and the coefficients of each path are each statistically significant (see Table 2 for details).

### 3.3. Path Coefficient of Structural Equation Model

The final fitted model shows a high degree of goodness of fit to the occupational population of the electronic manufacturing industry. The paths of measurement model has acceptable loadings, among which the standardized regression weight of suicide ideation to depression is 0.48 at minimum, and the loadings of other measurement paths are all above 0.50. The correlation coefficient between the latent variable job burden and capital is −0.68. The standardized regression weights from latent variable burden to depression and well-being are 0.19 and −0.18, respectively; i.e., once job burden increases by a unit, depression increases by 0.19 units, and well-being decreases by 0.17 units. Similarly, the standardized path loadings between capital and the two aspects are −0.34 and 0.31, respectively. With increase of capital, depression decreased gradually, and well-being increased gradually. In the personality characteristics, the susceptibility of job stress, ergasiomania and the characteristics of thinking of work all the time increase occupational pressure and reduce overall work satisfaction. The relationship between depression and well-being is negative, and the standardized covariance is −0.29 (see Figure 1).

### 3.4. Multi-Group Structural Equation Model Analyses

To explore different structural associations of individual characteristics, we used the hierarchical method of structural equation modelling. We adopted the method of Nested Model Comparisons to discuss influences of different characteristics on the model’s goodness of fit; then we analysed the model’s path coefficients according to different characteristics. We constrained the path between the three latent variables and depression and well-being. Results of Nested Model Comparisons show that gender, age, education level and marital status have no significant influence on model fit. The position variable, as test results of the hierarchical model, show that the model difference is of statistical significance (*p* = 0.033), but changes in AGFI, NNFI, IFI, RMSEA, and other parameters are slight, significantly less than 0.05. In general, results are quite comparable with those for the total sample. However, there are some differences regarding factor loadings of specific working conditions and personality on latent factors for each group. For example, for the variable of gender, the female group has significant loading (0.27) from burden to depression, while the male group shows significant loading (0.14) from personality to depression. Additionally, variables of marriage and education show different associations in burden to depression and well-being. Position buffers the personality’s effects (see Table 3).

### 3.5. The Structural Model’s Direct and Indirect Effects

To further confirm study hypotheses 1 and 2 and to explore the relationship between JBC and health outcomes, we conducted mediating effect analysis of the structural equation model. Results show that the direct effect of job burden on depression and well-being are 0.19 (0.09–0.29) and −0.18 (−0.29–−0.09), respectively, and the indirect effects were 0.06 (0.02–0.10) and −0.11 (−0.16–−0.07) through personality. Similarly, direct effect of capital to depression and well-being are −0.34 (−0.42–−0.26) and 0.31 (0.23–0.38), respectively. The indirect effect through the individual personality is −0.02 (−0.03–−0.01) and 0.03 (0.01−0.06) respectively. At the same time, the personality also directly affects depression and well-being. It can mediate the relationship between the two dimensions of job burden and capital and health outcomes (see Table 4).

## 4. Discussion

Generally speaking, the job burden-capital model is the reform of mainstream models. It avoids some of limitations of the JDC model or the ERI model. The JDC or the ERI model focuses on limited aspects of work-related stressors, and this might initially lead to ignorance of the impact on the occupational population’s health by other working conditions [20]. However, the burden-capital model, in terms of job burden, integrates occupational psychological demands and workload, making the assessment of extrinsic risking conditions more comprehensive than the “job demands” of the JDC model. That is, the working condition of job burden can be reflected more comprehensively in terms of stressors during work, thus expanding the scope of application to the occupational population. Similarly, in respect to the extrinsic stress protective factor—job capital, sense of control at work (e.g., job skills, autonomy), social support and social exchange (e.g., respect, fairness, identity, income) are included. In turn, this reflects, under a particular job burden level, the impact on occupational stress and even health damage’s occurrence or development by job capital. Notably also, compared with the job demand-resource model, the job burden-capital model has identified that the occupational population’s individual personalities are also an important intrinsic factor in occupational stress’s occurrence or development. The role of personality in the relationships of psychological working conditions and health outcomes (depression or well-being) has been simultaneously verified.

By reviewing theoretical models of occupational stress (e.g., job demand-control-social support model, effort-reward imbalance model), job burden-capital-personality model of occupational stress summarizes specific working conditions as follows: the dimension of job burden that increases risk of depression and reduces well-being, and the dimension of capital that reduces risk of depression and improves well-being. Individual characteristics play an intermediary role in the relationship between working conditions and health outcomes. The latent variable of job burden is mainly measured based on the following:

- Workload and psychological demands, such as over tasking, time pressure and complex operations, which might directly or indirectly increase risk of occupational stress’ occurrence and development;
- Capital, which mainly includes job autonomy, skills, social support, feedback, job stability and job prospects, such as personal job skills, job autonomy, fair treatment, future development, work stability, sense of identity, respect sense, income and social support;
- Dimension of personality that mainly involves the three typical characteristics of overwhelmed, laid-back, and postponing.

Analysis of sub-items’ internal consistency reliability show that selected variables or dimensions have good internal consistency. Hypothesis 1 proposed that job burden and capital can be directly associated with depression and well-being. Correlation and SEM analyses show that job burden positively correlates to depression, in other words, a higher burden is directly associated with greater depression and lower well-being. Similarly, increased capital significantly relates to risk of depression and increased well-being, which has negatively correlates with job burden [31]. Moreover, job burden and capital could also indirectly affect depression and well-being through personality. Indirect effects alter in different variables, of which job burden has stronger indirect effects than job capital. In other words, job burden influences personal characteristics more to affect occupational outcomes indirectly. 

Hypothesis 2 proposed that the individual personality might mediate the relationship between job burden-capital and depression-well-being. TaruFeldt’s work indicates that participants with low over-commitment as a certain personal characteristic with a set of attitudes reflecting excessive striving combined with a strong desire for approval [38] tend to score higher in well-being [39]. Individual personality, such as exaggerating their efforts beyond levels or exposing high demands at work too often, might reduce potential to recover from job demands and increase susceptibility to frustration [40]. In addition to direct influences on occupational outcomes, results show that individual personality could also mediate effects between job burden-capital and outcome of working status.

Analysis of multi-group SEM shows that the model has good applicability to different genders, ages, education levels, marital statuses and positions. According to good applicability, we can detect these variables differentiated effects. Results show that gender, marriage and education load significantly different coefficients of the path from job burden to depression, indicating that such variables act as adjusting roles for the association between these two aspects. In other words, single male employees with a low educational level might weaken health effects of depression caused by a job burden. Similarly, we found that the male gender is also a risk factor for depression by personality, and a single employee shows fewer preventive effects with decreasing burden.

The next deduction might be obtained through comparison of job burden and capital’s direct effect on outcome. That is, capital has a more obvious effect on depression and well-being; this result is similar to some existing research. In both the JDC model and the demand-resource model, job demands’ impacts on job strain could be buffered by job control/resources [12,41]. Additionally, the JDC neglected control’s role in affecting occupational outcomes until Bakker indicated that job resources are not only essential for dealing with job demands, but they have influencing effects in their own right as well [12]. In other words, job capitals either play an intrinsic motivational role because they promote employees’ growth, learning and development or play an extrinsic motivational role because they are helpful in achieving work goals [12,42]. This indicates that employees with adequate capitals for work tasks will probably be obtained and completed [43], consistent with either previous stress theory or our model. That is to say, job burden, a major source of tension, might play a role in depression and well-being, while capital is necessary to adjust health outcomes, work efficiency or motivation. Furthermore, the correlation coefficient indicates interactive effects between them in development of occupational outcomes [43]. 

In the job burden-capital model, psychological working conditions in different dimensions are now comparable; that is, workload versus psychological demand, effort versus control or social support can be compared and explained within a unified index under a common framework. Results of the measurement model by structural equation analysis have also tested this conclusion: the loading of each measurement dimension in the job burden-capital model is greater than 0.50, i.e., the contribution of corresponding stress factors to psychological working conditions is quite even, and thus comparable. This provides a reliable research platform for further exploring psychological working conditions’ impact on health. Based on this platform, this article has measured the extrinsic stress factor—job burden—and the extrinsic protection factor—job capital. Thus, it clarified the relationship between burden/capital and depression/health. That is, an increase in either psychological needs or working task requirements of job burden might directly damage health, while increase in capital might have protective effects on health. From another point of view, the result reflects the model’s flexibility in use. More specifically in the present study, different dimensions of stress have different roles in depression and health status, helping healthcare workers or enterprise managers identify populations at high risk of depression; for example, single male employees with low educational levels might weaken the health effects of depression caused by job burden. The application of the JBC model in stress study might deepen our understanding of depression’s health damage and help distinguish different stress characteristics among different occupational populations.

In the future, this model can be used to investigate occupational stress and its health impacts on a broader occupational scope and to explore its reliability and validity in different occupational groups. At the same time, it will be meaningful to improve the model’s measurement indicator system continuously and to ameliorate the theoretical framework for comprehensive reflection of work-related stress among occupational populations. Moreover, the model is helpful in detecting modifiable conditions such as skill, social support or even personality related to work, which employers could promote through professional training and culture construction. Thus, practical evidence for health intervention under the job burden–capital model must be illustrated in future studies, and the model’s reliability and validity remain to be further investigated before application in other language contexts.

## 5. Limitations

The JBC model emphasizes working conditions (i.e., job burden and capitals in evaluating depression and well-being). Measurement of these dimensions is mainly based on existing scales or targeted self-rated questions. Thus, which measurement could be consistent with the theory’s hypotheses is limited. However, these findings are clearly in line with previous studies and theories, and the related analyses have also shown high reliability of items. This study’s second limitation is its basis on self-report questionnaires, which might lead to subjective bias in the data. Nevertheless, our findings’ consistency with the theory, together with the acceptable sample size, suggests that common-method bias is not a major drawback of our study. Moreover, because of study samples’ limited availability, we collected information only from employees of the electronic manufacturing service industry in Hunan Province, possibly restricting our results’ generalizability. Lastly, the causal relationship between study variables (psychological working conditions and depression or well-being) cannot be evaluated with a cross-sectional design. Therefore, the theoretical model should be gradually revised, and psychological working conditions’ causal effects on health outcomes should be verified by future confirmatory studies.

## 6. Conclusions

This study focuses on development of a more systematic and comprehensive occupational stress–health outcomes model. Through analysis of structural equation model, mediation effects analysis and other statistical procedures, the following hypotheses have been tested: 1. Job burden and capital are directly associated with depression and well-being; 2. Personality can mediate the relationship among job burden, capital, depression and well-being; 3. For occupational groups, the theoretical model has general applicability among populations with different characteristics. Gender, marriage and education influence the association of model variables. Thus, the model provides more comprehensive insight into the relationship between occupational stress and its corresponding health outcomes.

## Figures and Tables

**Table 1:** Analysis of correlation and internal consistency of each dimension.

Dimensions/Items	$\bar{\chi }$ ± S	Cronbach’s α	1	2	3	4	5	6	7	8	9	10	11	12
1. Job burden	2.69 ± 0.71	0.863	1.00											
2. Workload	2.42 ± 0.83	0.839	0.874 *	1.00										
3. PD	2.97 ± 0.81	0.805	0.867 *	0.516 *	1.00									
4. Capital	3.50 ± 0.77	0.943	−0.495 *	−0.574 *	−0.284 *	1.00								
5. Autonomy	2.46 ± 0.98	0.812	−0.299 *	−0.358 *	−0.161 *	0.743 *	1.00							
6. Skills	3.32 ± 0.94	0.802	−0.296 *	−0.369 *	−0.144 *	0.803 *	0.579 *	1.00						
7. SS	3.66 ± 0.81	0.903	−0.329 *	−0.400 *	−0.171 *	0.777 *	0.512 *	0.725 *	1.00					
8. Feedback	3.96 ± 0.85	0.869	−0.528 *	−0.607 *	−0.310 *	0.878 *	0.499 *	0.638 *	0.610 *	1.00				
9. Stability	3.95 ± 1.01	0.754	−0.417 *	−0.482 *	−0.241 *	0.773 *	0.414 *	0.457 *	0.466 *	0.705 *	1.00			
10. Prospects	3.68 ± 1.11	0.750	−0.507 *	−0.551 *	−0.331 *	0.858 *	0.571 *	0.531 *	0.528 *	0.800 *	0.657 *	1.00		
11. Personality	2.52 ± 0.89	0.847	0.513 *	0.495 *	0.396 *	−0.415 *	−0.308 *	−0.227 *	−0.279 *	−0.411 *	−0.374 *	−0.394 *	1.00	
12. Depression	1.00 ± 0.55	0.889	0.366 *	0.416 *	0.218 *	−0.506 *	−0.321 *	−0.425 *	−0.489 *	−0.447 *	−0.379 *	−0.407 *	0.325 *	1.00
13. Well-being	3.10 ± 1.22	0.924	−0.451 *	−0.449 *	−0.336 *	0.516 *	0.418 *	0.394 *	0.405 *	0.443 *	0.371 *	0.460 *	−0.419 *	−0.450 *

Note: * *p* < 0.01; PD: psychological demands; SS: social support.

**Table 2:** The process of model fitting and the goodness of fit of structural models.

Dimensions/Models		$x^{2}$	df	χ^2^/df	AGFI	NNFI	IFI	RMSEA
M1	Original	851.09	19	44.79	0.775	0.832	0.886	0.165
	Modified	72.50	14	5.18	0.971	0.984	0.992	0.051
M2	Original	1001.02	41	24.42	0.836	0.864	0.899	0.120
	Modified	221.88	36	6.16	0.955	0.970	0.980	0.057
M3	Original	3026.44	167	18.12	0.791	0.804	0.828	0.103
	Modified	2308.52	163	14.16	0.836	0.849	0.871	0.090
M4	Original	2635.46	101	26.09	0.766	0.816	0.845	0.125
	Modified	1801.85	97	18.58	0.878	0.871	0.896	0.104
M5	Original	1766.66	164	10.77	0.863	0.888	0.903	0.078
	Modified	1018.57	160	6.37	0.919	0.938	0.948	0.058
M6	Original	1384.39	98	14.13	0.859	0.904	0.922	0.090
	Modified	522.61	94	5.56	0.944	0.967	0.974	0.053
M7	Original	2404.31	266	9.04	0.858	0.898	0.910	0.071
	Modified	1493.86	261	5.72	0.910	0.940	0.948	0.054
M8	Original	2325.27	265	8.78	0.861	0.901	0.913	0.069
	Modified	1397.05	260	5.37	0.915	0.945	0.952	0.052

*Notes*: the M1 model includes only job burden and capital; M2: add personal characteristics dimension and related path on the basis of M1; M3: include the three dimensions of job burden, capital, individual personality and depression, and add dimension to the depression path; M4: include the three dimensions of job burden, capital and individual personality and well-being, and add three dimensions to the well-being path; M5: add the relation path of three dimensions of job burden, capital and individual personality on the basis of M3; M6: add the interaction path of three dimensions of job burden, capital and individual personality on the basis of M4; M7: combine M5 with M6; M8: add the interaction paths of depression and well-being variables on the basis of M7; df: degree of freedom.

**Table 3:** Path Coefficients of the Structural Model in the Job Burden–capital Model of Depression and Well-Being for the Whole Sample and for the Multi-Groups Separately.

Variables		B to D	B to W	C to D	C to W	P to D	P to W	Model Test
Total Sample		0.19 *	−0.18 *	−0.34 *	0.31 *	0.11 *	−0.21 *	
Gender	Male	0.11	−0.22 *	−0.40 *	0.27 *	0.14 *	−0.21 *	*p* = 0.472
	Female	0.27 *	−0.14 *	−0.27 *	0.34 *	0.08	−0.20 *	
Marriage	Couple	0.25 *	−0.21 *	−0.33 *	0.25 *	0.10 *	−0.24 *	*p* = 0.192
	Single	0.07	−0.11	−0.38 *	0.39 *	0.13 *	−0.17 *	
Age	<25 years	0.21 *	−0.19 *	−0.28 *	0.24 *	0.11 *	−0.20 *	*p* = 0.805
	25–30 years	0.16 *	−0.17 *	−0.38 *	0.35 *	0.10	−0.21 *	
	>30 years	0.21 *	−0.19 *	−0.35 *	0.30 *	0.11 *	−0.21 *	
Education	<High school	0.08	−0.15 *	−0.24 *	0.15 *	0.10 *	−0.22 *	*p* = 0.100
	Junior high school	0.25 *	−0.23 *	−0.31 *	0.32 *	0.09	−0.15 *	
	College and above	0.13 *	−0.25 *	−0.49 *	0.31 *	0.10 *	−0.23 *	
Position	Assembly line	0.16 *	−0.18 *	−0.29 *	0.22 *	0.06	−0.17 *	*p* = 0.033
	Other production	0.30 *	−0.24 *	−0.19 *	0.24 *	0.19 *	−0.27 *	
	Logistical	0.20 *	−0.23 *	−0.46 *	0.36 *	0.06	−0.19 *	

**Note**: B = Burden; C = Capital; P = Personality; D = Depression; W = Well-being; * = *p* < 0.05.

**Table 4:** Direct and indirect effects of the structural model.

Dimensions/Effect	Mediator		Outcomes			
	Personality	*p* Value	Depression	*p* Value	Well-Being	*p* Value
Direct Effect						
Job burden	0.52(0.41–0.63)	<0.001	0.19 (0.09–0.29)	<0.001	−0.18 (−0.29–−0.09)	<0.001
Capital	−0.14 (−0.24–−0.04)	0.010	−0.34 (−0.42–−0.26)	<0.001	0.31 (0.23–0.38)	<0.001
Character	—	—	0.11 (0.03–0.19)	0.006	−0.21 (−0.28–−0.14)	<0.001
Indirect Effect						
Job burden	—	—	0.06 (0.02–0.10)	0.005	−0.11 (−0.16–−0.07)	<0.001
Capital	—	—	−0.02 (−0.03–−0.01)	0.009	0.03 (0.01–0.06)	0.007
